# Interdependence of JAK-STAT and MAPK signaling pathways during EGF-mediated HTR-8/SVneo cell invasion

**DOI:** 10.1371/journal.pone.0178269

**Published:** 2017-05-25

**Authors:** Ankita Malik, Rahul Pal, Satish Kumar Gupta

**Affiliations:** 1 Reproductive Cell Biology Laboratory, National Institute of Immunology, New Delhi, Delhi, India; 2 Immunoendocrinology Laboratory, National Institute of Immunology, New Delhi, Delhi, India; University of Hyderabad, INDIA

## Abstract

Invasion of trophoblast cells is spatio-temporally regulated by various cytokines and growth factors. In pregnancy, complications like preeclampsia, shallow invasion of trophoblast cells and low amounts of epidermal growth factor (EGF) have been reported. In the present study, regulatory mechanisms associated with EGF-mediated invasion in HTR-8/SVneo trophoblastic cells have been delineated. Treatment of HTR-8/SVneo cells with EGF (10 ng/ml) led to eight fold increase (p < 0.05) in invasion. Increased invasion of HTR-8/SVneo cells by EGF was associated with an increase in phosphorylation of ERK½. In addition, significant phosphorylation of STAT1 (ser 727) and STAT3 (both tyr 705 and ser 727 residues) was also observed, accompanied by a decrease in total STAT1. Inhibition of ERK½ phosphorylation by U0126 (10 μM) led to a significant decrease in EGF-mediated invasion with simultaneous decrease in the phosphorylated forms of STAT3 and STAT1. Decrease in total STAT1 was also reversed on inhibition of ERK½. Interestingly, inhibition of STAT3 by siRNA led to a significant decrease in EGF-mediated invasion of HTR-8/SVneo cells and phosphorylation of STAT1, but it did not have any effect on the activation of ERK½. On the other hand, inhibition of STAT1 by siRNA, also led to a significant decrease in the EGF-mediated invasion of HTR-8/SVneo cells, showed concomitant decrease in ERK½ phosphorylation and STAT3 phosphorylation at ser 727 residue. These results suggest cross-communication between ERK½ and JAK-STAT pathways during EGF-mediated increase in invasion of trophoblast cells; phosphorylation at ser 727 residue of both STAT3 and STAT1 appears to be critical.

## Introduction

Inadequate or shallow trophoblast invasion associated with poor spiral artery remodeling has been observed in the placentae of women with preeclampsia (PE), intrauterine growth restriction (IUGR) or late sporadic miscarriage [1–5], while excessive trophoblast invasiveness can cause placental accreta [6]. A variety of cytokines and growth factors such as interleukin-6 (IL-6), interleukin-11(IL-11), hepatocyte growth factor (HGF), leukemia inhibitory factor (LIF) and epidermal growth factor (EGF) are known to increase migration and invasion of trophoblast cells [7]. While factors like tumor necrosis factor alpha (TNFα) and interferon gamma (IFNγ) are known to decrease the migration and invasion of trophoblast cells [8]. A fine balance between these invasion-promoting and -inhibiting factors in the uterine microenvironment determines placentation by controlling trophoblast cell invasion.

EGF (53 amino acid polypeptide) is known to increase invasion of trophoblast cells [9] and is secreted by early human placenta and uterine glands. EGF and its receptor are localized on the cytotrophoblast cells at 4–5 weeks after fertilization and augments their proliferation. At 6–12 weeks of conception, EGF and its receptor are present on syncytiotrophoblasts, and EGF signaling stimulates human chorionic gonadotropin (hCG) and human placental lactogen (hPL) secretion without affecting cytotrophoblast proliferation [10, 11]. Lower levels of EGF in plasma and urine have been reported in women with preeclampsia and IUGR respectively [12–13]. In preeclampsia patients, p110/EGFR (a truncated epidermal growth factor receptor isoform) has also been reported to be elevated [14]. Recent studies have also associated single nucleotide polymorphism in EGF gene with preeclampsia and low birth weight babies [15]. EGF is known to stimulate motility and invasion of trophoblast cells by activation of urokinase plasminogen activator (uPA), plasminogen activator inhibitor (PAI)-1, matrix metaloproteinases (MMP) -2, -9 by PI3-K, Akt as well as activation of both p38 and p44/42 mitogen-activated protein kinase (MAPK) signaling [16–20]. Further, transcription factor p53 controls EGF-induced increase in MMP-2 levels in JAR choriocarcinoma cell line [21] and silencing of transcription factor AP-2α reduces EGF induced increase in invasion in primary extravillous trophoblast cells (EVTs) and in the SGHPL-5 trophoblast cell line [22]. EGF activates these downstream signaling pathways by binding to the receptor tyrosine kinases of the human EGF receptor family, which has four members [23].

STAT3 signaling is indispensable for induction and regulation of invasion of trophoblast as well as cancer/tumor cells [24]. It is also reported that STAT3 and its phosphorylated form are significantly decreased in EVT, villous trophoblast and entire placentas in patients with preeclampsia [25]. As STAT3 plays a very important role in promoting invasion of trophoblast cells, it is important to study the effect of EGF treatment on activation of the JAK-STAT pathway. Signaling pathways seldom work in isolation and multiple pathways are activated after cytokine or growth factor treatment of cells. Thus, it is important to understand how EGF-mediated upregulated pathways interact or compensate for each other when either activated or inhibited.

Keeping the above in view, the aim of this study was to delineate the relative role of MAPK and JAK-STAT signaling pathways during EGF-mediated invasion of trophoblast cells. HTR-8/SVneo cells derived from human first trimester placental explant cultures immortalized by SV40 large T antigen [26] closely resemble physiological phenotype of isolated first trimester EVT cells and has been used extensively to study trophoblast invasion [18, 19, 27–32]. Further, HTR-8/SVneo cells shows similar responses as primary EVTs to TGF-β while choriocarcinoma trophoblast cell lines like JEG-3 and JAR failed to show similar response as EVTs due to loss of Smad3 [33]. Further, IL-11 is known to decrease trophoblast outgrowth of EVTs in which GRP78 plays a crucial role, which shows similar expression in HTR-8/SVneo cells after IL-11 treatment [34]. We have also previously shown that IL-11 treatment of HTR-8/SVneo cells led to decrease in HTR-8/SVneo cells invasion while increased JEG-3 invasion [35]. Thus, it is reasonable to conclude that HTR-8/SVneo cells respond similarly to the physiological ligands as primary trophoblast cells while JEG-3 and JAR choriocarcinoma cell lines fail to do so due to loss of intermediate signaling molecules. Hence, we used HTR-8/SVneo as a model to study the signaling pathways associated with EGF-mediated invasion.

## Materials and methods

### Cell culture

The HTR-8/SVneo cell line (kindly provided by Prof. P. K. Lala, Queens’s University, Kingston, ON, Canada) was maintained in a 1:1 mixture of Dulbecco’s modified Eagle medium and Ham’s F-12 medium (Sigma-Aldrich Inc, St Louis, MO, USA) supplemented with 10% heat-inactivated fetal bovine serum (FBS; Gibco^®^, Life Technologies, CA, USA) and Pen-Strep-Amphosol (100 U/ml penicillin; 100 μg/ml streptomycin; 0.25 μg/ml amphotericin B) an antibiotic antimycotic cocktail (Biological Industries, Kibbutz beit Haemek, Israel) under humidified conditions of 5% CO_2_ in air at 37°C. The cells were passaged at 70–80% confluency and maintained in Geneticin (G418) sulphate (Amersham Life Sciences, Cleveland, OH, USA) at 75 μg/ml every third passage [26].

### Invasion assay

Invasion assay was performed as described previously [35]. Briefly, 0.8 μm transwell inserts (Greiner Bio, Kremsműnster, Austria) were coated with 50 μl of matrigel matrix (1 μg/ml, BD Biosciences, San Jose, CA, USA) and incubated in a 24-well plate at 37°C overnight to solidify. Approximately 0.1 X 10^6^ HTR-8/SVneo cells were seeded in the upper chamber of the transwell in 150 μl of 1:1 DMEM + Ham’s F-12 medium supplemented with 1% FBS along with an optimized concentration of EGF (10 ng/ml; Life Technologies, Carlsbad, CA, USA). The lower chamber was filled with 300 μl of medium with same concentration of EGF as in upper chamber. After 24 h incubation at 37°C in 5% humidified CO_2_, excess of cells and matrigel from the upper chamber were aspirated and cleaned with moist cotton swab. The cells on the lower side of the membrane were fixed with chilled methanol at 4°C for 5–10 min and stained with 0.2 μM Hoechst 33342 (Thermo Fisher, Massachusetts, USA) nuclear dye at 37°C for 5 min. The membrane was visualized under fluorescent phase contrast microscope ECLIPSE TE2000-E (Nikon, Tokyo, Japan) under oil immersion and cells were counted on the complete membrane using Image pro-plus software (developed by Media Cybernetics, Maryland, USA). The value of untreated control was taken as one and fold change was calculated by dividing number of cells on treated transwell insert membrane by the number of cells on untreated control transwell insert membrane.

### Preparation of whole cell extract and Western blot

Cells (~0.1 X 10^6^) seeded in six well plates (24 h prior) were starved of FBS for 4 h and then treated with EGF (10 ng/ml) for 10, 30 and 60 min in serum free medium. Medium was aspirated for each time point and cells were lysed in 50 μl/well of RIPA buffer (20 mM Tris HCl, 10% glycerol, 0.2 mM EDTA, 0.137 M NaCl and 1% NP-40) supplemented with a protease and phosphatase inhibitor cocktail (Roche Diagnostics GmbH, Mannheim, Germany). The lysate was freeze-thawed in liquid nitrogen thrice to ensure complete cell lysis followed by centrifugation at 12,000 g for 10 min at 4°C and the supernatant was collected. Amount of protein in each sample was estimated using a BCA protein estimation kit (Thermo Scientific, Massachusetts, USA) as per the manufacturer’s instructions. Equivalent volume for each sample containing 40 μg of protein were denatured at 95°C for 5 min, resolved on SDS-PAGE and transferred to a nitro-cellulose membrane (Mdi, Ambala Cantt., India) as described previously [35]. The membrane was blocked with 5% BSA in Tris buffered saline (TBS) (Sigma-Aldrich Inc.) for 1 h at room temperature. One 5 min washing in TBS was given and membranes were incubated with primary antibodies against p44/42ERK½ total, P-p44/42ERK½, actin, STAT3 total, p-STAT3 (tyr 705), p-STAT3 (ser 727), STAT1 total, p-STAT1 (ser 727) (Cell Signaling Technologies, Massachusetts, USA) diluted in TBS-T (0.1% Tween) with 1% BSA at recommended dilutions at 4°C overnight. After three 10 min washing in TBST, membranes were incubated in secondary antibodies (anti-mouse HRPO for STAT3 total, actin or anti-rabbit HRPO, Cell Signaling Technologies) at 1:2000 dilution for 1 h at room temperature. Blots were developed after three washings in TBST by chemiluminescence HRP substrate (Merck Millipore, Massachusetts, USA). To measure individual protein expression levels, intensities of specific bands corresponding to the signaling proteins were measured using Image J software [36].

### Inhibition of ERK½ phosphorylation by U0126

HTR-8/SVneo cells (0.1 X10^6^/well) seeded in 6-well plates on the previous day were serum starved for 4 h. Cells were kept in 10 μM U0126 (Cell Signaling Technologies) as per manufacturer’s instructions for 2 h to inhibit ERK½ phosphorylation. Cells were then processed for matrigel invasion assay in presence or absence of EGF (10 ng/ml). Inhibition of ERK½ phosphorylation was confirmed by Western blot for each experiment.

### Silencing of STAT3 and STAT1 expression by siRNA

HTR-8/SVneo cells (0.1 X10^6^/well) were seeded in 6-well plates. For STAT3 silencing, custom-made sense and antisense nucleotide sequences (sense- UGUUCUCUAUCAGCACAAUtt and antisense- AUUGUGCUGAUAGAGAACAtt; Qiagen HP flexible design siRNA STAT3 alpha) and for STAT1 silencing, a smart pool of 3 different siRNAs (Santa Cruz Biotechnology, Texas, USA) were used. Cells were washed in Opti-MEM (Gibco Thermo Fisher Scientific, Massachusetts, USA) and 750 μl of fresh Opti-MEM was added to each well. STAT3, STAT1, scrambled control siRNA (optimized concentrations of 40 pmol for STAT3 and 20 pmol for STAT1) were mixed with Opti-MEM to make a total volume of 125 μl. In a separate tube, 6 μl lipofectamine 3000 (Thermo Fisher) was mixed with 119 μl of Opti-MEM and incubated for 5 min at room temperature. Both the solutions were mixed (1:1) and incubated for 10 min at room temperature and added drop wise in respective wells. After 6 h incubation, 1:1 DMEM + Ham’s F12 medium supplemented with 10% FBS was added to the wells. After 24 h of silencing, medium was replaced by adding fresh 1.5 ml 1:1 DMEM + Ham’s F12 medium supplemented with 10% FBS to the wells and incubated at 37°C in presence of humidified 5% CO_2_ for 48 h. At 72 h after silencing, cells were processed for invasion assay, Western blot and qRT-PCR. The silencing following transfection was confirmed by qRT-PCR as well as Western blot.

### Statistical analysis

All experiments were performed thrice and the results were expressed as the mean of values obtained in the three experiments ± SEM. For each set of experiments of invasion assay, Western blot (densitometric analysis) and qRT-PCR, the statistical analysis was carried out by comparing means of the experimental set (after normalizing) with the control sets by one-way ANOVA. A value of p ≤ 0.05 was considered to be statistically significant.

## Results

### Treatment of HTR-8/SVneo cells with EGF led to increase in invasiveness associated with activation of ERK½, STAT1 and STAT3

**Fig 1:**
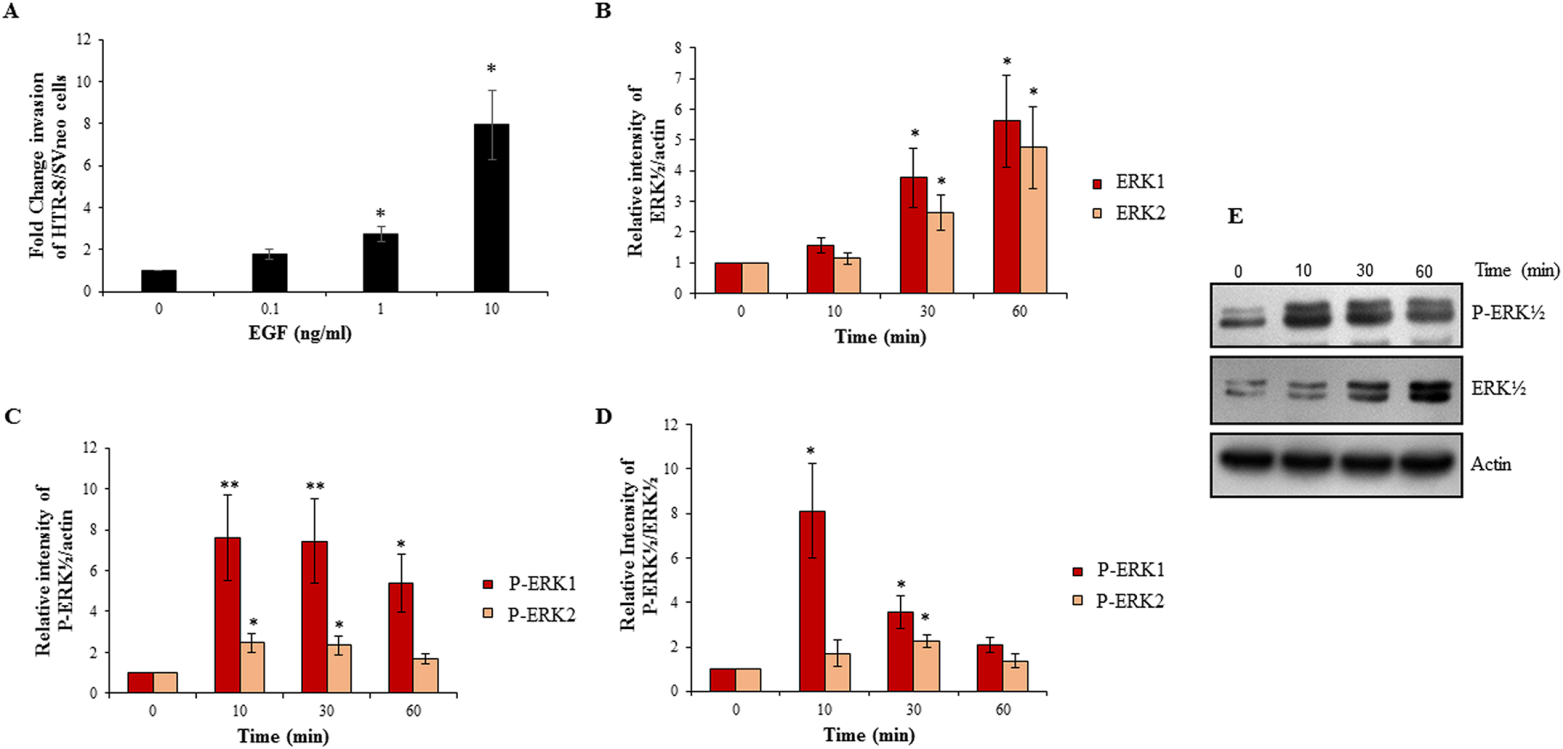
EGF treatment of HTR-8/SVneo cells increases invasion and activates ERK½ signaling pathway. Cells were treated with varying concentrations of EGF (0.1, 1, 10 ng/ml). Panel A shows fold change in invasion of EGF-treated cells with respect to untreated naive control cells. Values are expressed as mean ± SEM of three independent experiments performed in duplicates. Further experiments were performed by treating cells with optimized EGF concentration (10 ng/ml) for varying time periods (10, 30 and 60 min) followed by Western blot analysis to determine activation of ERK½ as described in *Materials and Methods*. Panel B represents the densitometric plot of relative increase in total ERK 1 and 2 in EGF treated cells with respect to untreated control as compared with actin. Panels C and D represent the densitometric plots showing the relative increase in phosphorylated ERK 1 and 2 in EGF treated cells with respect to untreated control as compared to actin and total ERK 1 and 2 respectively. Panel E shows representative blots from one of the three independent experiments. The data in Panels B, C and D is represented as mean ± SEM of three independent experiments and band intensities of phosphorylated and total ERK½ were normalized with respect to loading control during densitometric analysis. *p < 0.05 and **p < 0.01 with respect to untreated control cells.

The effect of varying EGF concentrations (0, 0.1, 1, 10 ng/ml) on invasiveness of HTR-8/SVneo cells was measured in a matrigel invasion assay. EGF led to an increase in the number of invading cells in a concentration-dependent manner and EGF at 10 ng/ml led to the highest increase (~8 fold) in invasiveness (Fig 1A); hence, further experiments were performed at this concentration. To rule out that EGF-mediated increase in invasion of the cells is not due to increase in proliferation, BrdU cell proliferation assay following EGF treatment of HTR-8/SVneo cells was performed. No significant increase in cell proliferation was observed (S1 Fig). Subsequently, HTR-8/SVneo cells were treated with EGF (10 ng/ml) for varying time periods (0, 10, 30, 60 min) and cell lysates were processed for assessment of activated signaling pathways by Western blot as described in *Materials and Methods*. EGF treatment led to significant increase in total ERK½ (at 30 min; Fig 1B and 1E). Significant increase in ERK½ phosphorylation was seen at 10 and 30 min; and also ERK1 phosphorylation at 60 min with respect to actin as loading control (Fig 1C and 1E). Further, significant increase in phosphorylation of ERK1 was observed by 10 min and ERK2 by 30 min with respect to total ERK½ (Fig 1D and 1E). As compared to basal levels of phosphorylated ERK1 and 2, in EGF treated cells the increase in levels of ERK1 phosphorylation was more than ERK2 phosphorylation (Fig 1C, 1D and 1E).

**Fig 2:**
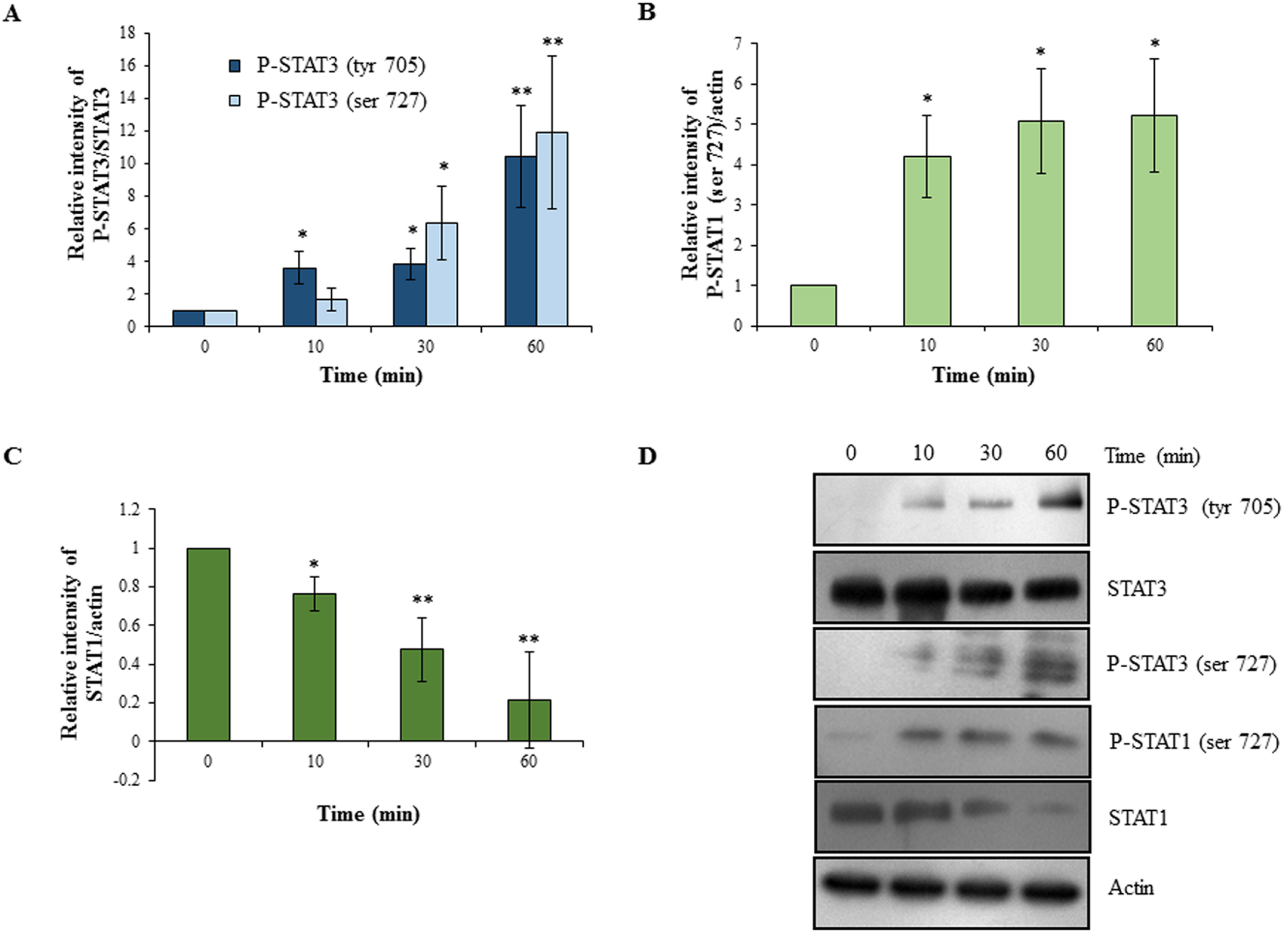
Activation of STAT3 and STAT1 dependent signaling following EGF treatment of HTR-8/SVneo cells. HTR-8/SVneo cells were treated with EGF (10 ng/ml) for varying time periods followed by Western blot analysis to determine activation of STAT1 and STAT3 as described in *Materials and Methods*. Panel A represents the densitometric plot showing relative increase in phosphorylated STAT3 (ser 727 and tyr 705) levels in EGF treated cells with respect to untreated control as compared to total STAT3. Panel B represents the densitometric plot showing relative increase in phosphorylated STAT1 (ser 727) levels in EGF treated cells with respect to untreated control as compared to actin. Panel C represents the densitometric plot showing relative decrease in total STAT1 levels in EGF treated cells with respect to untreated control as compared to actin. Panel D shows representative blots from one of the three independent experiments. The data in Panels A, B and C is represented as mean ± SEM of three independent experiments and band intensities of phosphorylated and total STAT1 & STAT3 were normalized with respect to loading control during densitometric analysis. *p < 0.05 and **p < 0.01 with respect to untreated control cells.

Following EGF treatment of HTR-8/SVneo cells, statistically significant increase in activation of STAT3 (phosphorylation both at tyr 705 and ser 727 residues) was also observed (Fig 2A and 2D). However, no significant changes in total STAT3 were observed at all the time points studied (Fig 2D). There was significant increase in the STAT1 phosphorylation at ser 727 as early as at 10 min, which continued till 60 min (Fig 2B and 2D). Interestingly, the amount of total STAT1 was reduced by 80% in the cells after 60 min of EGF treatment (Fig 2C and 2D).

### Inhibition of ERK½ phosphorylation led to decrease in invasion of HTR-8/SVneo cells with concomitant inhibition of STAT1 and STAT3 phosphorylation

**Fig 3:**
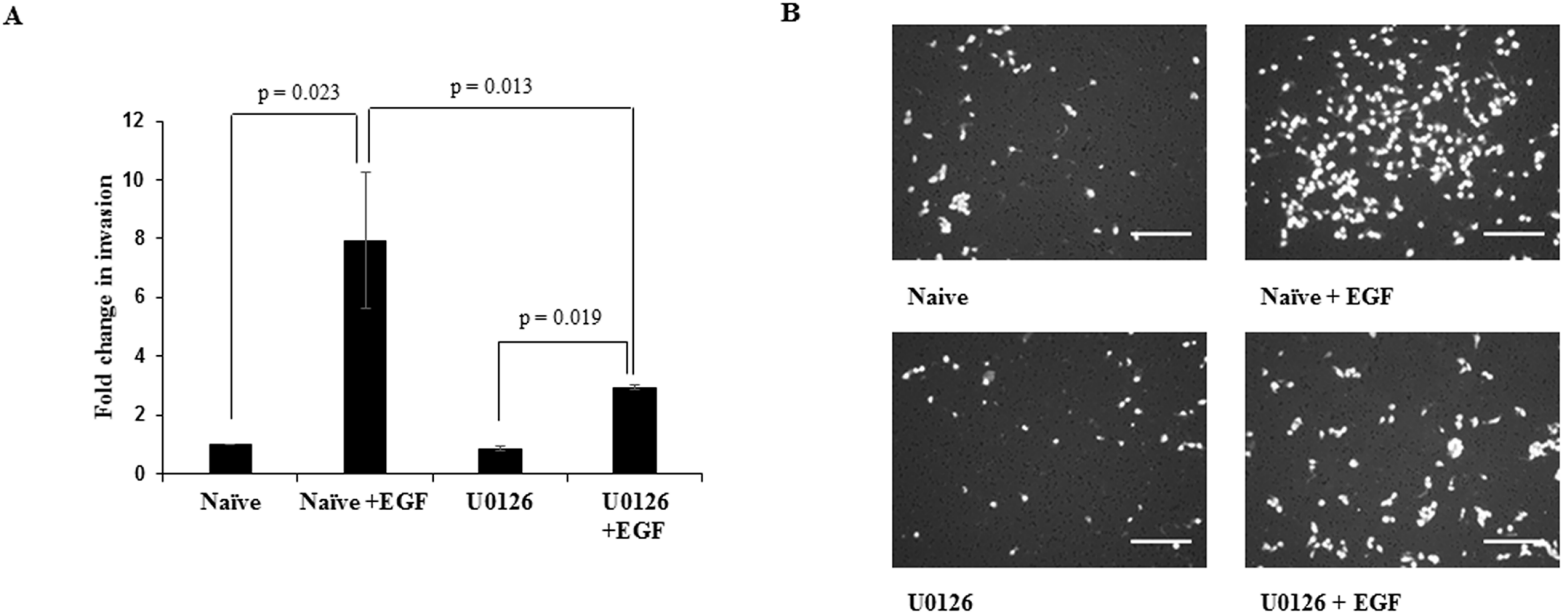
Effect of U0126 pre-treatment of HTR-8/SVneo cells on invasion in presence or absence of EGF. Following U0126 pre-treatment of HTR-8/SVneo cells, invasion assay was performed either in presence or absence of EGF (10 ng/ml) as described in *Materials and Methods*. Panel A shows relative fold change in invasion of varying treatment groups (EGF treated, U0126 pre-treated and U0126 pre-treated cells treated with EGF) as compared to the untreated naïve HTR-8/SVneo cells. Values are expressed as mean ± SEM of three independent experiments performed in duplicates. Panel B has representative photographs of invading cells in various treatment groups (naïve, EGF treated, U0126 pre-treated and U0126 pre-treated cells with EGF treatment) on 0.8 μm pore size transwell membranes as observed under microscope after processing for invasion assay. Scale bar represents 5 μm.

**Fig 4:**
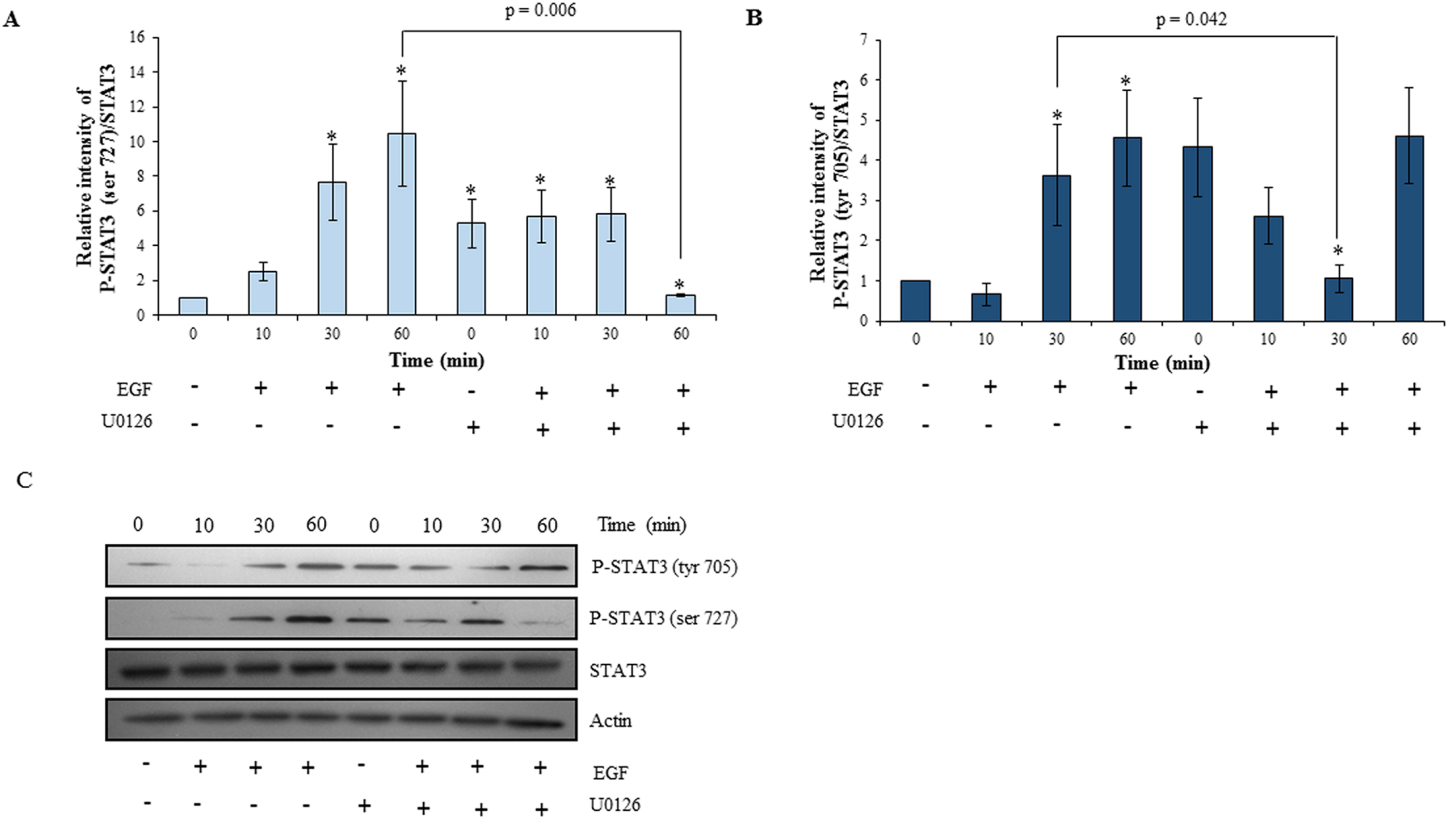
Effect of inhibition of ERK½ signaling in HTR-8/SVneo cells on EGF-mediated STAT3 activation. HTR-8/SVneo cells with or without pretreatment of U0126 for 2 h, were treated with EGF (10 ng/ml) for varying time points (10, 30 and 60 min) and Western blotting was performed as described in *Materials and Methods*. Panels A and B represent the densitometric profiles of P-STAT3 ser 727 and tyr 705 levels as compared to STAT3 total at 10, 30 and 60 min after EGF treatment of HTR-8/SVneo cells (with or without U0126 pre-treatment) with respect to untreated control respectively. Panel C shows the representative blots from one of the three independent experiments. The data is shown as mean ± SEM of three independent experiments and band intensities were normalized with respect to loading control during densitometric analysis. *p < 0.05 with respect to untreated control cells.

**Fig 5:**
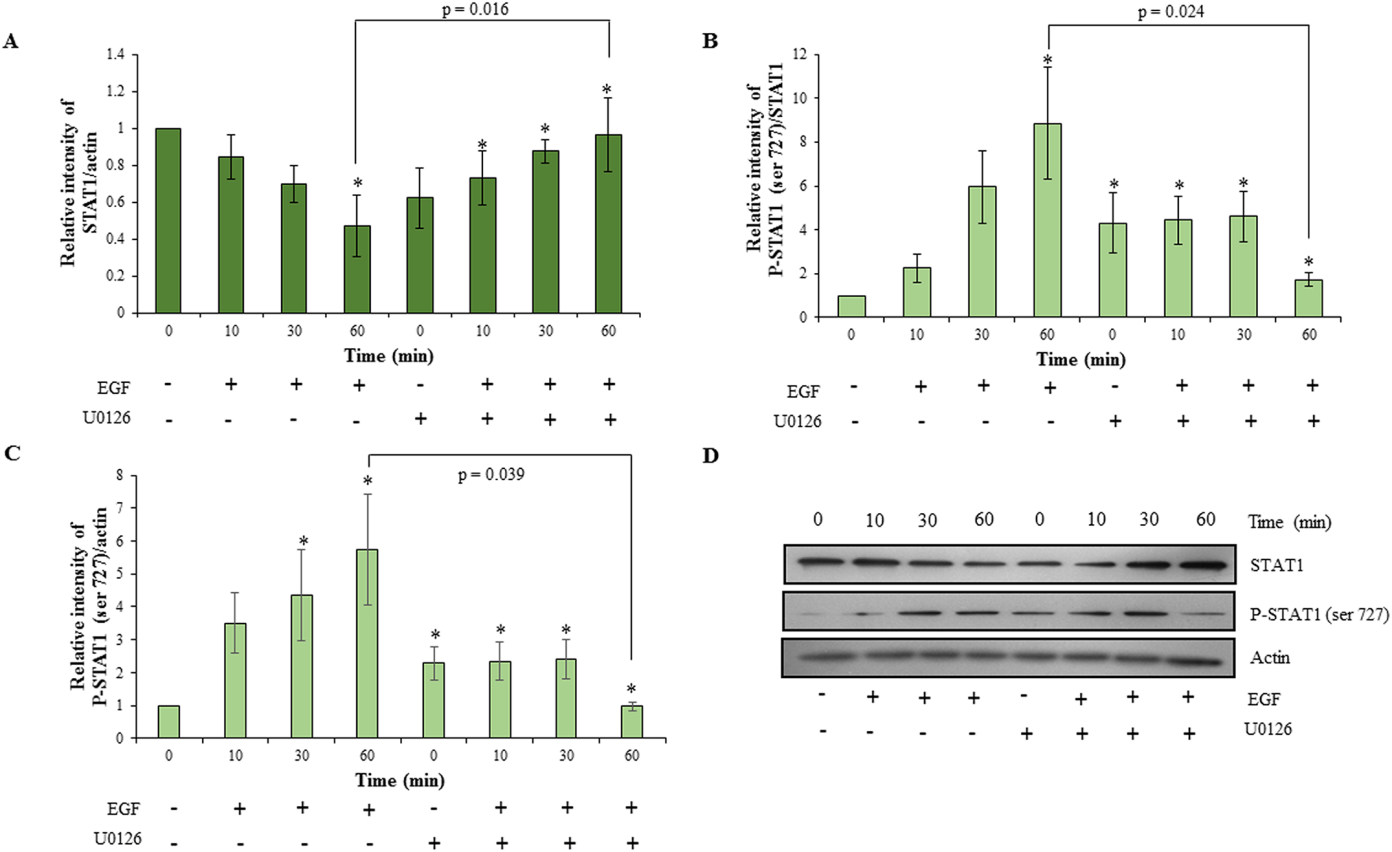
Effect of inhibition of ERK½ signaling in HTR-8/SVneo cells on EGF-mediated STAT1 activation. HTR-8/SVneo cells with or without pretreatment of U0126 for 2 h, were treated with EGF (10 ng/ml) for varying time points (10, 30 and 60 min) and Western blotting was performed as described in *Materials and Methods*. Panel A represents the densitometric profile of total STAT1 levels as compared to actin at 10, 30 and 60 min after EGF treatment of HTR-8/SVneo cells (with or without U0126 pre-treatment) with respect to untreated control respectively. Panels B and C represent the densitometric profiles of P-STAT1 ser 727 levels as compared to STAT1 and actin respectively. Panel D shows representative blots from one of the three independent experiments. The data is shown as mean ± SEM of three independent experiments and band intensities were normalized with respect to loading controls during densitometric analysis. *p < 0.05 with respect to untreated control cells.

To understand the role of ERK½ activation on EGF-mediated invasion and to probe the possibility of existence of cross-talk among the various activated signaling pathways, the MAPK phosphorylation inhibitor U0126 was used to inhibit ERK½ phosphorylation. After 4 h of serum starvation, HTR-8/SVneo cells were pre-treated with 10 μM U0126. Cells were further treated with an optimized concentration of EGF (10 ng/ml) and processed for the invasion assay or for Western blot. U0126 pre-treatment had no effect on basal invasiveness of HTR-8/SVneo cells; however, it significantly inhibited EGF-induced increase in invasion as compared to naïve cells treated with EGF (Fig 3). The inhibition of EGF-mediated increase of HTR-8/SVneo cells invasion by pre-treatment with U0126 was not complete, as it showed significant increase in invasiveness as compared to U0126 pre-treated cells without any EGF treatment (Fig 3). To rule out the role of apoptosis in decreasing invasion, cell viability was assessed by SYTOX red flow cytometry; no significant cell death was observed after U0126 treatment (S2 Fig). U0126 significantly decreased EGF-induced ERK½ phosphorylation (S3 Fig). Basal levels of the phosphorylated forms of STAT3 and STAT1 in U0126 pre-treated HTR-8/SVneo cells were observed to be higher, but increase in their phosphorylation on EGF treatment at various time points as compared to basal levels of STAT3 (tyr 705 and ser 727) and STAT1 (ser 727) was not observed (Figs 4 and 5). STAT3 at the ser 727 residue showed significant loss of increased basal phosphorylation after U0126 pre-treatment at 60 min after EGF treatment, which was equivalent to the phosphorylation observed in the untreated naïve cells (Fig 4A and 4C). STAT3 phosphorylation of tyr 705 was significantly reduced at 30 min of EGF treatment in U0126 pre-treated cells (Fig 4B and 4C). U0126 pre-treatment was able to rescue STAT1 from degradation (Fig 5A and 5D). Loss of increased basal phosphorylation of STAT1 ser 727 residue was also observed, which was equivalent to the phosphorylation observed in the untreated naïve cells (Fig 5B, 5C and 5D).

### Silencing of STAT3 by siRNA led to decrease in invasion of HTR-8/SVneo cells without affecting phosphorylation of ERK½ but decreased phosphorylation of STAT1 ser 727

**Fig 6:**
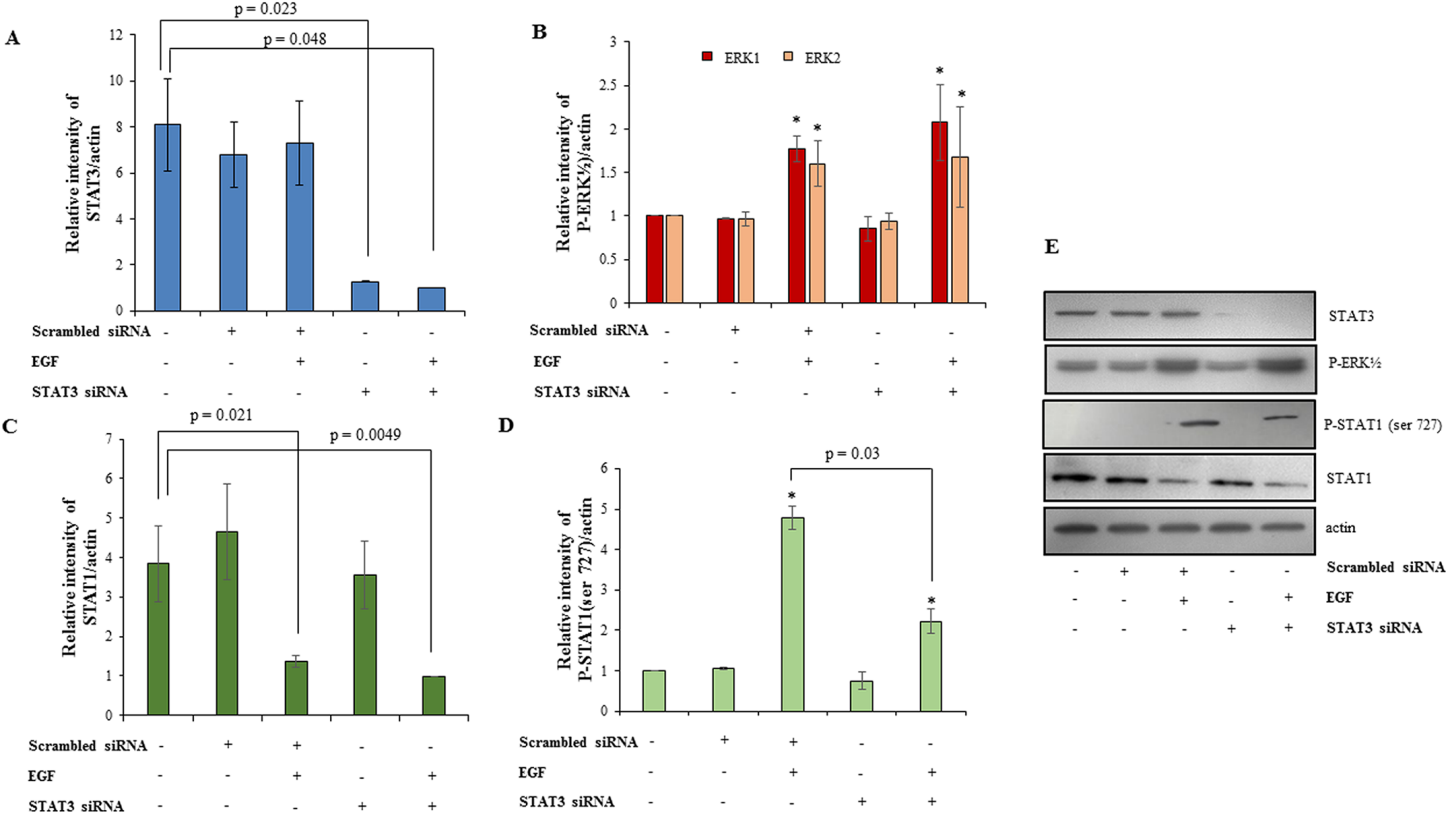
Effect of STAT3 silencing on phosphorylation of ERK½ and STAT1 in presence/absence of EGF treatment. STAT3 was silenced in HTR-8/SVneo cells using siRNA as described in *Materials and Methods*. The naïve cells, scrambled siRNA and STAT3 siRNA transfected cells were treated with EGF (10 ng/ml) for 60 min. Panel A shows densitometric plot of total STAT3 levels as compared to actin either in presence or absence of EGF treatment to confirm silencing of STAT3. Panels B, C and D represent densitometric profiles of phosphorylated ERK½, total STAT1 and phosphorylated STAT1 ser727 respectively, as compared to actin in naïve cells, scrambled and STAT3 siRNA treated cells in presence or absence of EGF. Panel E shows the representative blots from one of the three independent experiments. The data is shown as mean ± SEM of three independent experiments and band intensities were normalized with respect to loading controls during densitometric analysis. *p < 0.05 with respect to untreated control cells.

**Fig 7:**
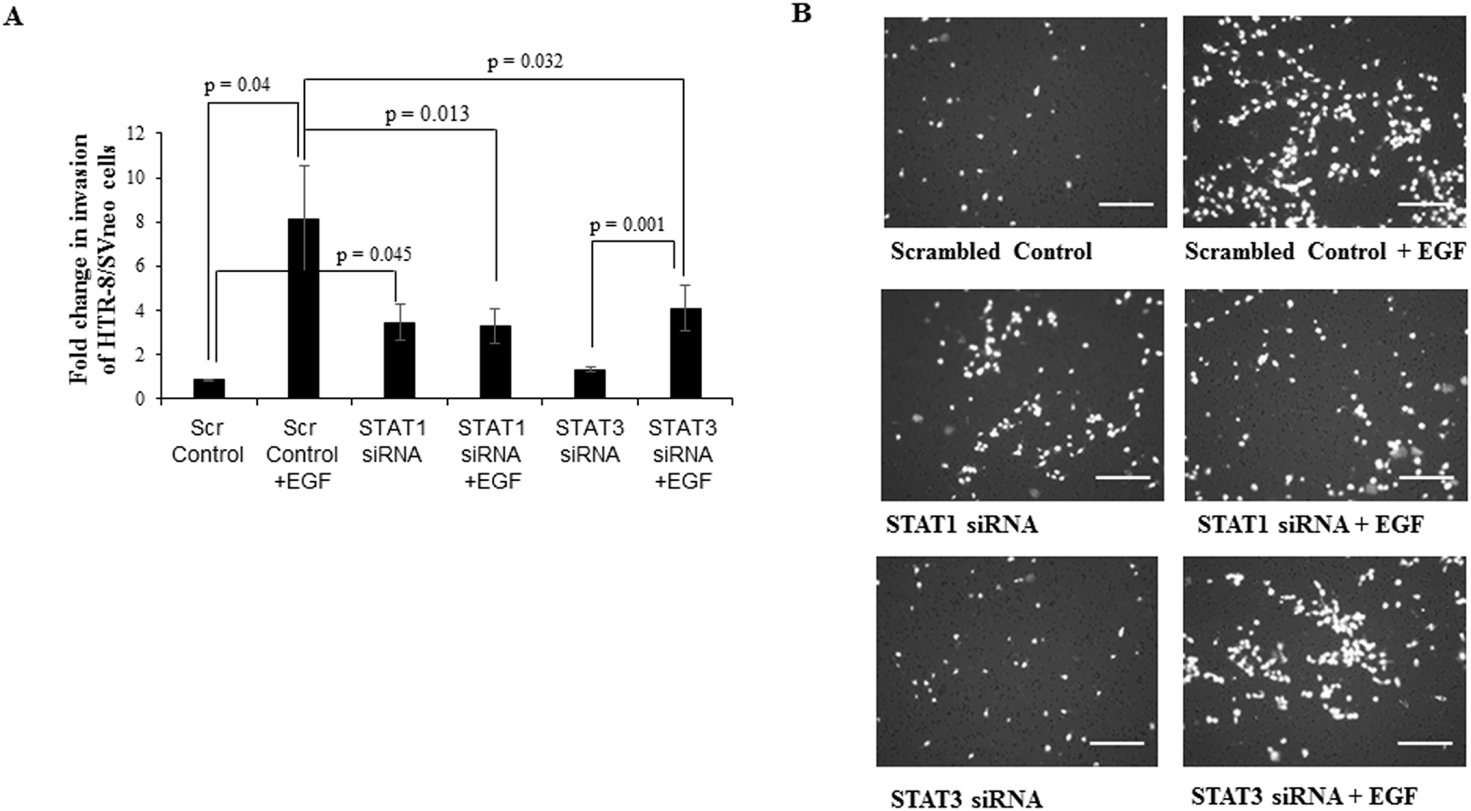
Effect of STAT3 and STAT1 silencing on invasion in presence or absence of EGF. Following STAT1 and STAT3 silencing in HTR-8/SVneo cells using respective siRNA, invasion assay was performed either in presence or absence of EGF (10 ng/ml) as described in *Materials and Methods*. Panel A shows relative fold change in invasion following EGF treatment of scrambled siRNA, STAT1 siRNA and STAT3 siRNA transfected cells as compared to the untreated HTR-8/SVneo cells. Values are expressed as mean ± SEM of three independent experiments performed in duplicates. Panel B has representative photographs of invading cells in EGF treated scrambled siRNA, STAT1 siRNA and STAT3 siRNA transfected cells and the respective controls on 0.8 μm pore size transwell membranes as observed under microscope (10X objective) after processing for invasion assay. Scale bar represents 5 μm.

STAT3 expression in the HTR-8/SVneo cells was silenced using siRNA as described in *Materials and Methods*. Silencing led to 80% reduction in STAT3 expression as verified by Western blot (Fig 6A and 6E). To understand the significance of EGF-induced STAT3 activation, scrambled and STAT3 siRNA treated cells were treated with EGF (10 ng/ml) for 24 h along with appropriate controls. STAT3 silencing led to a significant reduction in the EGF-induced increased invasiveness of HTR-8/SVneo cells, but had no effect on the basal invasion rate (Fig 7). Further, to investigate whether STAT3 silencing affected the phosphorylation of ERK½ and STAT1 degradation, lysates from STAT3 silenced cells were processed for Western blot after EGF treatment for 60 min. It was observed that the amount of phosphorylated ERK½ and total STAT1 were not significantly different in EGF-treated STAT3 silenced cells as compared to that of EGF-treated scrambled siRNA transfected cells (Fig 6B, 6C and 6E). However, a decrease in STAT1 phosphorylation at the ser 727 residue in STAT3 silenced cells was observed (Fig 6D and 6E).

### Silencing of STAT1 by siRNA led to decrease in invasion of HTR-8/SVneo cells accompanied by decrease in phosphorylation of ERK½ and STAT3 ser 727

**Fig 8:**
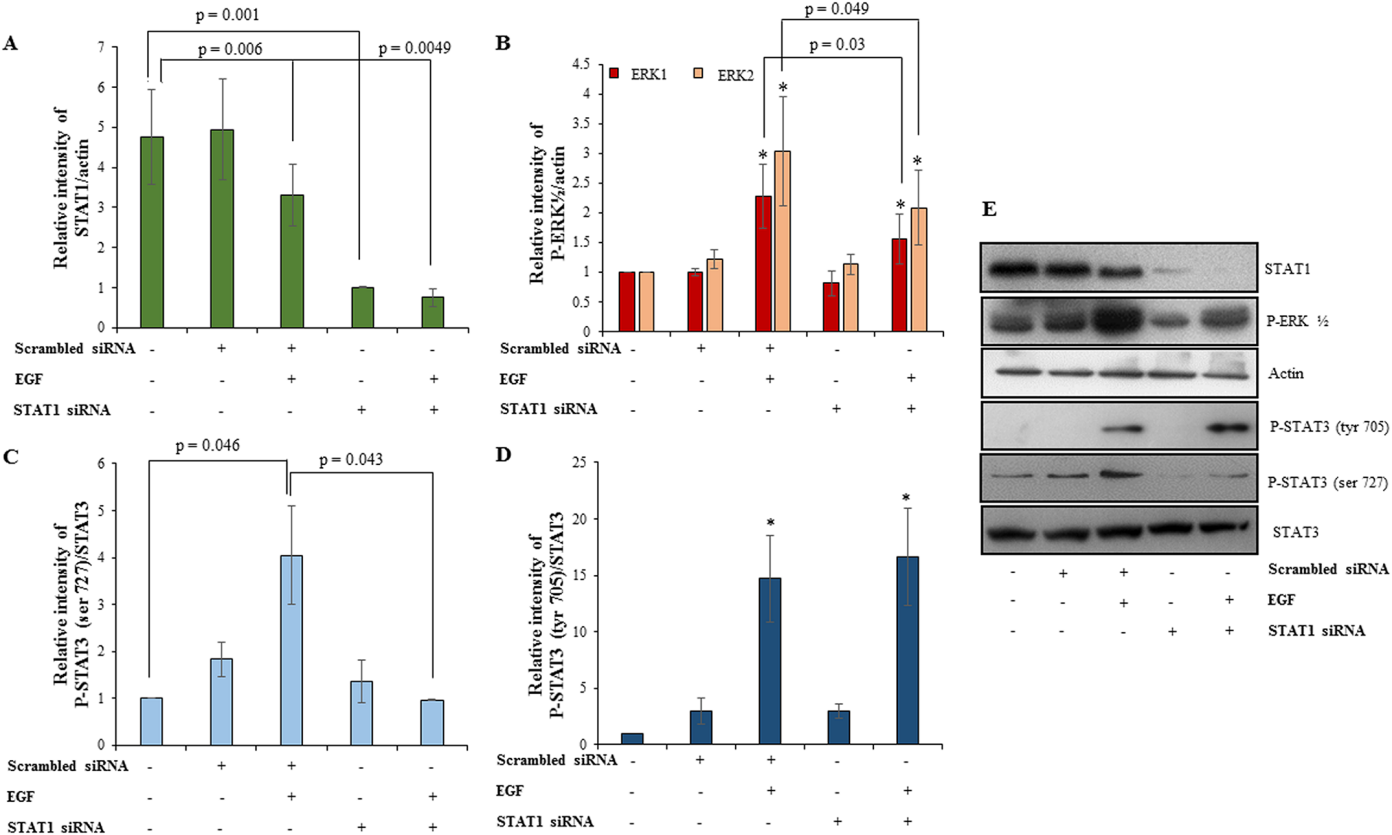
Effect of STAT1 silencing on phosphorylation of ERK½ and STAT3 in presence/absence of EGF treatment. STAT1 was silenced in HTR-8/SVneo cells using siRNA as described in *Materials and Methods*. The naïve cells, scrambled siRNA and STAT1 siRNA transfected cells were treated with EGF (10 ng/ml) for 60 min. Panel A shows densitometric plot of total STAT1 levels as compared to actin in presence or absence of EGF treatment to confirm silencing of STAT1 by Western blot. Panels B, C and D represent densitometric profiles of phosphorylated ERK½ as compared to actin, phosphorylated STAT3 at ser727 and tyr 705 as compared to total STAT3 respectively in naïve cells, scrambled and STAT1 siRNA treated cells in presence or absence of EGF. Panel F show representative blots from one of the three independent experiments. The data is shown as mean ± SEM of three independent experiments and band intensities were normalized with respect to loading control during densitometric analysis. *p < 0.05 with respect to untreated control cells.

STAT1 silencing performed as described in *Materials and Methods*, led to significant decrease in total amounts of STAT1 both at transcript (S4 Fig) and protein levels (Fig 8A). To understand the significance of EGF-induced STAT1 activation and its degradation, STAT1 silenced cells were treated with EGF in invasion assay for 24 h. STAT1 silencing led to significant basal increase in the invasiveness of HTR-8/SVneo cells as compared to the scrambled siRNA transfected control (Fig 7). However, significant reduction in invasiveness of EGF treated STAT1 silenced HTR-8/SVneo cells was observed as compared to the EGF-mediated increased invasiveness of HTR-8/SVneo cells treated with scrambled siRNA (Fig 7). Treatment of STAT1 siRNA transfected HTR-8/SVneo with EGF failed to show any significant increase in the invasiveness as compared to STAT1 silenced cells without any treatment with EGF (Fig 7). Further, the effect of STAT1 silencing on EGF-induced ERK½ and STAT3 phosphorylation was investigated. These studies revealed decrease in the amount of phosphorylated ERK½ and P-STAT3 ser 727, whereas P-STAT3 tyr 705 remained unaffected in EGF-treated STAT1 silenced cells as compared to EGF-treated scrambled siRNA transfected cells (Fig 8B, 8C, 8D and 8E).

## Discussion

**Fig 9:**
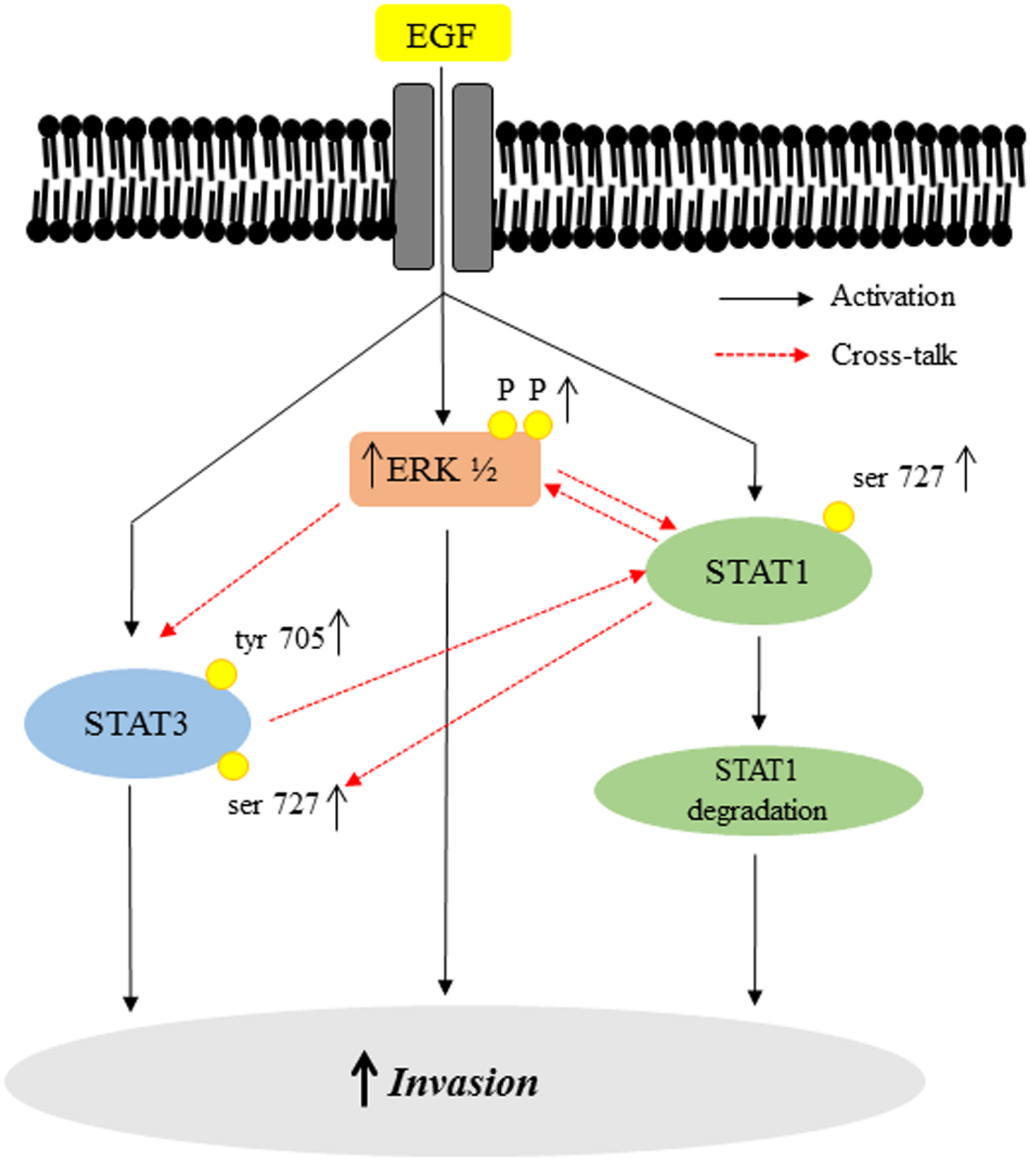
Schematic representation of the EGF-mediated activation of the downstream signaling pathways and their cross-talk. Binding of EGF to the HTR-8/SVneo cells induces increase in phosphorylation of ERK½, STAT1 ser 727 and STAT3 at both tyr 705 and ser 727 residues. It also led to decrease in total STAT1. These changes may be activating transcription of various invasion promoting genes leading to increase in invasion of HTR-8/SVneo cells subsequent to EGF treatment. By using a MAPK inhibitor, inhibition of EGF-induced ERK½ phosphorylation led to down-regulation of activation of STAT3 (at tyr 705 and ser 727) and STAT1 (ser 727) as well as STAT1 degradation in HTR-8/SVneo trophoblast cells. However, inhibition of STAT3 by siRNA only affects STAT1 ser 727 phosphorylation. On the other hand, STAT1 silencing by siRNA led to significant reduction in phosphorylation of ERK½ and STAT3 ser 727 without affecting STAT3 phosphorylation at tyr 705.

Understanding the signaling pathways that are relevant in regulating invasiveness of trophoblast cells is critical for understanding the molecular basis of pregnancy related complications like preeclampsia and IUGR. In patients suffering from preeclampsia and IUGR, deciphering the role of EGF in trophoblast invasion and downstream signaling might help better understanding of the significance of reported lower levels of EGF in these conditions [12, 13]. Although it is already known that the MAPK and PI3K pathways are involved in regulation of EVT migration in response to EGF, it is not clear whether JAK-STAT pathway is additionally involved. Recently, STAT5 activation after EGF treatment has been shown to increase the proliferation and viability of trophoblast cells [37]. In the preset study, using HTR-8/SVneo trophoblast cell line, a dose-dependent increase in invasiveness was observed upon EGF-treatment, which was associated with the activation of ERK½, STAT3 (ser 727 and tyr 705) and STAT1 (ser 727), as summarized in Fig 9. It has been shown that EGF increases proliferation of trophoblast cells [37]. However, we did not observe an increase in the proliferation of HTR-8/SVneo cells (S1 Fig) when treated with EGF, which may be due to the differences in the concentration of EGF employed (100 ng/ml *versus* 10 ng/ml used in present study). Tyrosine phosphorylation of STATs and their subsequent SH2 domain mediated homo/hetero dimerization results in translocation to the nucleus and is therefore essential for their biological activity. Serine phosphorylation is a general requirement for many resident nuclear transcription factors [37–40]. STAT1 and STAT3 ser 727 phosphorylation is important for their function as nuclear transcription factors [41]. Inhibition of STAT1 and STAT3 ser 727 phosphorylation reduces its factor-induced transcriptional activity [42]. Many signals are known to exclusively activate serine phosphorylations of STATs without activating tyrosine phosphorylation [43, 44]. Thus, exclusive serine phosphorylation might be a way of the system to prime STATs for an alternate and different transcriptional response in the cell. Differential STAT phosphorylations achieved by different ligands (IL-6, IL-11, INF γ, EGF), even when they might be controlling the same physiological response like trophoblast invasion, point towards the discretion and control exercised by receptors on downstream cell signaling activated in a particular cell type.

The ERK½ (serine/threonine kinase) is known to target conserved serine residues on several STATs (such as STAT1a, STAT3 and STAT4) [45]. It was observed that inhibition of ERK½ by U0126 in HTR-8/SVneo cells resulted in concomitant inhibition of EGF-induced increase in STAT1 and STAT3 phosphorylation at ser 727 along with inhibition of degradation of total STAT1 (Figs 4 and 5). These results suggest that there is a communication between ERK½, STAT1 and STAT3 during EGF-mediated HTR-8/SVneo cell invasion (Fig 9). Further, higher basal levels of phosphorylated STAT1 (ser 727) and STAT3 (both ser 727 and tyr 705) were observed after U0126 pre-treatment (Figs 4 and 5), with no effect on the cell viability (S2 Fig). Hence, this increased basal phosphorylation of STAT1 and STAT3 might be one of the way by which cell prepares to cope with inhibition of the MAPK pathway, a pathway important for cell survival and proliferation [45]. While, increased basal phosphorylation of STAT3 ser and tyr residues after U0126 treatment has been reported previously [46], the current study found that this increased basal phosphorylation had no effect on the invasiveness of HTR-8/SVneo cells (Fig 3). Absence of the increasing trend in phosphorylation with time of STAT3 (tyr 705 and ser 727) and STAT1 (ser 727), as compared to basal level in U0126 pre-treated cells, suggests that phosphorylation of STAT1 and STAT3 is also relevant in EGF-mediated increase in invasiveness of HTR-8/SVneo cells (Figs 3–5).

Taking into account many reports that stress the significance of STAT3 in regulating invasion in trophoblast cells [8, 47, 48] and the observed activation of STAT3 phosphorylation at both tyr 705 and ser 727 after EGF treatment, we further performed STAT3 silencing studies to see its effect on invasiveness and other signaling pathways. STAT3 silencing had no effect on basal invasiveness of HTR-8/SVneo cells but compromised EGF-induced increase in invasion (Fig 7). Further, STAT3 silencing had no effect on EGF-induced ERK½ phosphorylation or STAT1 degradation but STAT1 ser 727 phosphorylation was reduced (Fig 6). Hence, it can be concluded that STAT3 activation has unambiguous role in EGF-mediated invasion of trophoblast cells. Further, STAT1 silencing was also performed to ascertain its role in EGF-induced increased invasion of HTR-8/SVneo cells. STAT1 silencing increased basal invasiveness of the HTR-8/SVneo cells (Fig 7), which might be due to active negative regulation by STAT1 of invasion promoting proteins in absence of any extracellular signal as non-phosphorylated STATs are still able to function as transcription factors and alter gene expression [49]. After STAT1 silencing, EGF treatment was not able to further increase invasiveness of HTR-8/SVneo cells and it was comparable to STAT1 silenced cells without EGF treatment, which may be due to inability of EGF to further reduce already low levels of STAT1 in the STAT1 silenced cells. Significant reduction in phosphorylation of ERK½ and STAT3 at ser 727 was observed in STAT1 silenced cells after EGF treatment, which can possibly be due to absence of phosphorylated STAT1 ser 727 in STAT1 silenced cells (Fig 8). Similar, reduced ERK½ phosphorylation after STAT1 silencing and inhibition of STAT1 binding by Fludarabine has been observed previously [50], though the mechanism has not been elaborated. STAT1 and STAT3 phosphorylated forms can form heterodimers with each other [51]. The above results suggest that phosphorylation of STAT1 ser 727 may be responsible for STAT3 ser 727 phosphorylation or its stabilization by formation of a heterodimer and vice versa after EGF treatment. However, no significant changes were observed in STAT3 phosphorylation at tyr 705 after EGF treatment of STAT1 silenced cells as compared to EGF treated scrambled siRNA transfected cells (Fig 8). It is thus important to further elaborate on mechanism of how STAT1 and STAT3 silencing affect each other’s phosphorylation status during factor-induced signaling in cell systems. Thus, it can be concluded from these observations that reduction in EGF-induced ERK½ phosphorylation and ser 727 phosphorylation of STAT3 after STAT1 silencing might be responsible for no further increase in EGF-mediated invasiveness of STAT1 silenced HTR-8/SVneo cells.

Inhibition of ERK½ phosphorylation as well as STAT3 and STAT1 silencing in HTR-8/SVneo cells individually were not able to completely inhibit EGF-mediated increased invasion of HTR-8/SVneo cells. Partial, though significant inhibition of EGF-mediated invasion due to abrogation of individual pathways suggests that the activated pathways after EGF treatment are capable of compensating for each other, but this compensation is not at the same level as when all pathways are functional. Thus, it can be suggested that activation of MAPK and JAK-STAT pathways after EGF treatment are interdependent on each other and there is a cross-talk between both pathways which may play an important role during EGF-mediated increase in invasion of the HTR-8/SVneo cells. As inhibition of ERK½ phosphorylation as well as silencing of STAT1 and STAT3 affect phosphorylation of STAT1/STAT3 at ser 727, it suggests that phosphorylation at ser 727 residue of both STAT1 and STAT3 is critical in EGF-mediated increase in invasion of HTR-8/SVneo cells. As this study used transformed HTR-8/SVneo trophoblast cell line, it is important to perform key experiments in primary EVT cells or primary tissue sections to corroborate the findings. Low levels of EGF and reduced STAT3 phosphorylation are implicated in preeclampsia and IUGR. Therefore, these studies if expanded using primary EVT cells from women with preeclampsia will help in understanding whether low invasiveness observed in this condition is due to intrinsic defects in the downstream signaling or purely a consequence of low levels of EGF.

## Supporting information

S1 Fig Proliferation of HTR-8/SVneo cells with or without EGF treatment. Absorbance at 450 nm in EGF treated and untreated control cells in comparison to unstained cells by BrdU proliferation assay performed as per the manufacturer’s instructions (Millipore, Massachusetts, USA). Both control and EGF treated cells had similar ELISA readings.
(PDF)

S2 Fig Cell viability of naïve, EGF treated, U0126 and U0126+EGF treated cells. HTR-8/SVneo cells (0.1 X 10^6^/well) grown overnight in 6-well plates followed by serum starvation for 4 h, were treated with EGF, U0126 and U0126+EGF for 2 h. These cells were trypsinized and re-suspended in saline with working concentration of 5 μM SYTOX red. Appropriate unstained control and stained untreated control were taken. Percent total stained cells (dead cells) were calculated by flow Cytometry. Graph panels show percent stained cells (blue) as compared to unstained cells (red) for stained control, EGF, U0126, U0126+EGF treated groups. The above table shows the percent values of the stained cells.
(PDF)

S3 Fig Inhibition of ERK½ phosphorylation by U0126. Representative blots showing effect of U0126 pre-treatment on phosphorylation of ERK½ with or without EGF treatment in HTR-8/SVneo cells.
(PDF)

S4 Fig Transcript levels of STAT1 after silencing. Bar graph represents transcript levels of STAT1 mRNA by qRT-PCR in naïve, scrambled and STAT1 siRNA transfected cells either in presence or absence of EGF.
(PDF)
