# Supplementary material for: Interdependence of JAK-STAT and MAPK signaling pathways during EGF-mediated HTR-8/SVneo cell invasion
Source: PLoS One. 2017 May 25;12(5):e0178269. doi: 10.1371/journal.pone.0178269 (PMC5444796; doi:10.1371/journal.pone.0178269)
Supplement: S1 Fig — Absorbance at 450 nm in EGF treated and untreated control cells in comparison to unstained cells by BrdU proliferation assay performed as per the manufacturer’s instructions (Millipore, Massachusetts, USA). Both control and EGF treated cells had similar ELISA readings. (PDF) [file pone.0178269.s001.pdf]

S1 Fig

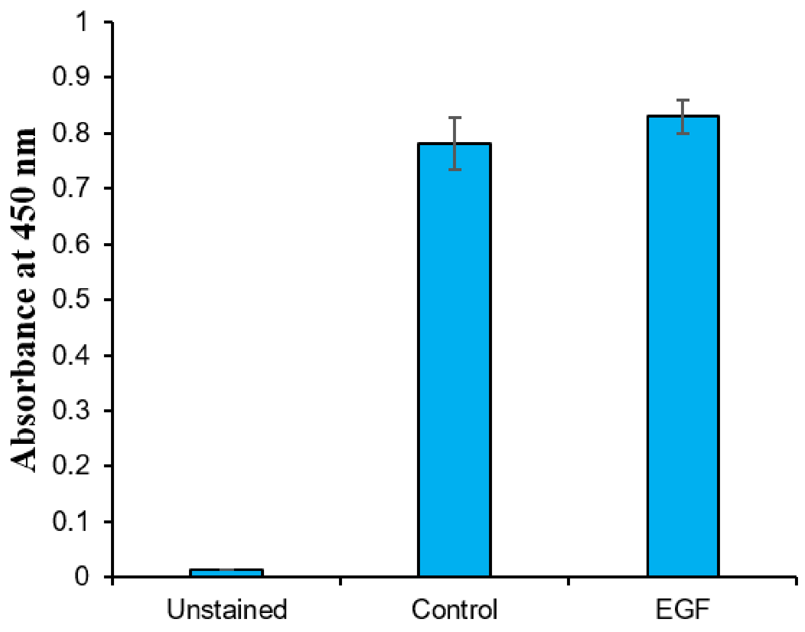

S1 Fig: Proliferation of HTR-8/SVneo cells with or without EGF treatment. Absorbance at 450 nm in EGF treated and untreated control cells in comparison to unstained cells by BrdU proliferation assay performed as per the manufacturer's instructions (Millipore, Massachusetts, USA). Both control and EGF treated cells had similar ELISA readings.
