# Supplementary material for: Interdependence of JAK-STAT and MAPK signaling pathways during EGF-mediated HTR-8/SVneo cell invasion
Source: PLoS One. 2017 May 25;12(5):e0178269. doi: 10.1371/journal.pone.0178269 (PMC5444796; doi:10.1371/journal.pone.0178269)
Supplement: S2 Fig — HTR-8/SVneo cells (0.1 X 106/well) grown overnight in 6-well plates followed by serum starvation for 4 h, were treated with EGF, U0126 and U0126+EGF for 2 h. These cells were trypsinized and re-suspended in saline with working concentration of 5 μM SYTOX red. Appropriate unstained control and stained untreated control were taken. Percent total stained cells (dead cells) were calculated by flow Cytometry. Graph panels show percent stained cells (blue) as compared to unstained cells (red) for stained control, EGF, U0126, U0126+EGF treated groups. The above table shows the percent values of the stained cells. (PDF) [file pone.0178269.s002.pdf]

S2 Fig

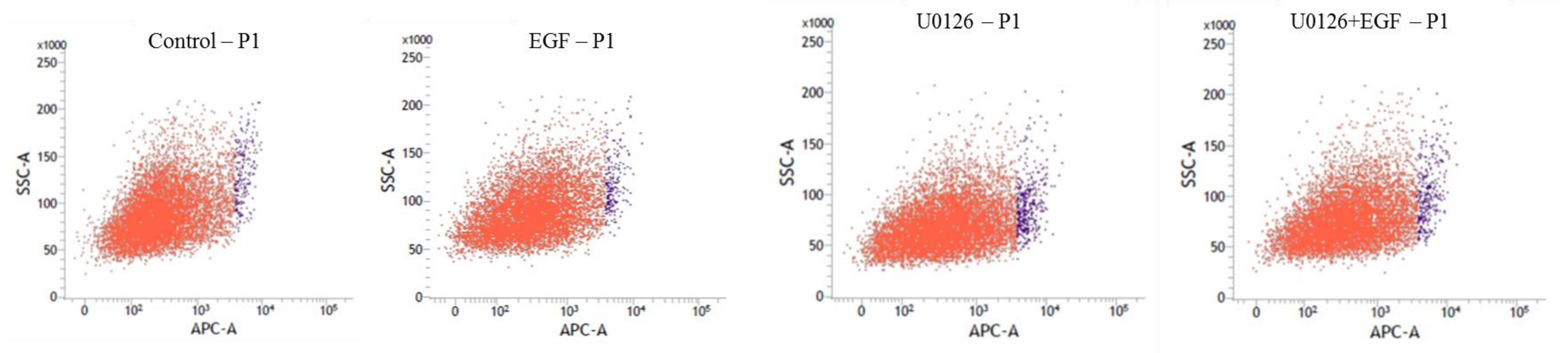

Sample	FSC-A mean	SSC-A Mean	% Total Stained (Dead cells)	% Parent	% Grandparent
Unstained Control	97,342	139.295	0.04	0.05	0.04
Stained Control	170,140	130,577	2.12	2.34	2.12
EGF treated	173,505	118,569	2.04	2.31	2.04
U0126 treated	171,601	89,679	3.67	4.07	3.67
U0126 + EGF treated	173,203	102,563	3.06	3,56	3.06

S2 Fig: Cell viability of naïve, EGF treated, U0126 and U0126+EGF treated cells. HTR-8/SVneo cells (0.1 X 10^6/well) grown overnight in 6-well plates followed by serum starvation for 4 h, were treated with EGF, U0126 and U0126+EGF for 2 h. These cells were trypsinized and re-suspended in saline with working concentration of 5 µM SYTOX red. Appropriate unstained control and stained untreated control were taken. Percent total stained cells (dead cells) were calculated by flow Cytometry. Graph panels show percent stained cells (blue) as compared to unstained cells (red) for stained control, EGF, U0126, U0126+EGF treated groups. The above table shows the percent values of the stained cells.
