# Supplementary material for: Interdependence of JAK-STAT and MAPK signaling pathways during EGF-mediated HTR-8/SVneo cell invasion
Source: PLoS One. 2017 May 25;12(5):e0178269. doi: 10.1371/journal.pone.0178269 (PMC5444796; doi:10.1371/journal.pone.0178269)
Supplement: S3 Fig — Representative blots showing effect of U0126 pre-treatment on phosphorylation of ERK½ with or without EGF treatment in HTR-8/SVneo cells. (PDF) [file pone.0178269.s003.pdf]

S3 Fig

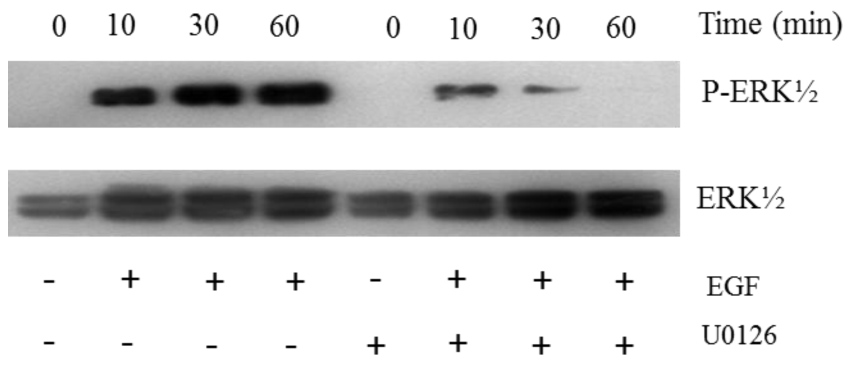

S3 Fig: Inhibition of ERK½ phosphorylation by U0126. Representative blots showing effect of U0126 pre-treatment on phosphorylation of ERK½ with or without EGF treatment in HTR-8/SVneo cells.
