# Supplementary material for: Interdependence of JAK-STAT and MAPK signaling pathways during EGF-mediated HTR-8/SVneo cell invasion
Source: PLoS One. 2017 May 25;12(5):e0178269. doi: 10.1371/journal.pone.0178269 (PMC5444796; doi:10.1371/journal.pone.0178269)
Supplement: S4 Fig — Bar graph represents transcript levels of STAT1 mRNA by qRT-PCR in naïve, scrambled and STAT1 siRNA transfected cells either in presence or absence of EGF. (PDF) [file pone.0178269.s004.pdf]

S4 Fig

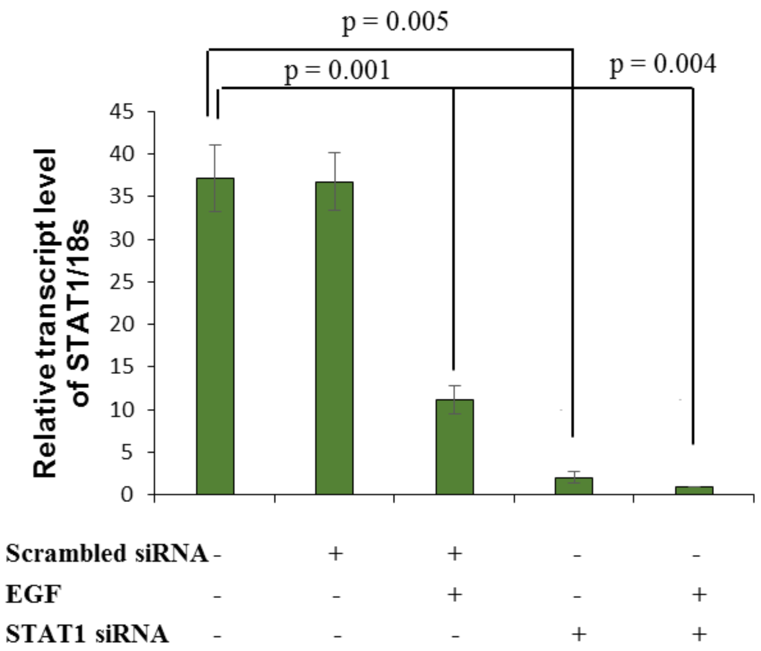

S4 Fig: Transcript levels of STAT1 after silencing. Bar graph represents transcript levels of STAT1 mRNA by qRT-PCR in naïve, scrambled and STAT1 siRNA transfected cells either in presence or absence of EGF.
